# Dissimilatory nitrogen reduction in intertidal sediments of a temperate estuary: small scale heterogeneity and novel nitrate-to-ammonium reducers

**DOI:** 10.3389/fmicb.2015.01124

**Published:** 2015-10-14

**Authors:** Helen Decleyre, Kim Heylen, Carl Van Colen, Anne Willems

**Affiliations:** ^1^Laboratory of Microbiology (LM-UGent), Department of Biochemistry and Microbiology, Ghent UniversityGhent, Belgium; ^2^Marine Biology Research Group, Department of Biology, Ghent UniversityGhent, Belgium

**Keywords:** ammonification, nitrate reducers, isolation, marine environment, intertidal sediments

## Abstract

The estuarine nitrogen cycle can be substantially altered due to anthropogenic activities resulting in increased amounts of inorganic nitrogen (mainly nitrate). In the past, denitrification was considered to be the main ecosystem process removing reactive nitrogen from the estuarine ecosystem. However, recent reports on the contribution of dissimilatory nitrate reduction to ammonium (DNRA) to nitrogen removal in these systems indicated a similar or higher importance, although the ratio between both processes remains ambiguous. Compared to denitrification, DNRA has been underexplored for the last decades and the key organisms carrying out the process in marine environments are largely unknown. Hence, as a first step to better understand the interplay between denitrification, DNRA and reduction of nitrate to nitrite in estuarine sediments, nitrogen reduction potentials were determined in sediments of the Paulina polder mudflat (Westerschelde estuary). We observed high variability in dominant nitrogen removing processes over a short distance (1.6 m), with nitrous oxide, ammonium and nitrite production rates differing significantly between all sampling sites. Denitrification occurred at all sites, DNRA was either the dominant process (two out of five sites) or absent, while nitrate reduction to nitrite was observed in most sites but never dominant. In addition, novel nitrate-to-ammonium reducers assigned to *Thalassospira, Celeribacter*, and *Halomonas*, for which DNRA was thus far unreported, were isolated, with DNRA phenotype reconfirmed through *nrfA* gene amplification. This study demonstrates high small scale heterogeneity among dissimilatory nitrate reduction processes in estuarine sediments and provides novel marine DNRA organisms that represent valuable alternatives to the current model organisms.

## Introduction

The rate of terrestrial nitrogen input has more than doubled over the past century, mostly through fossil fuel combustion and increased use of agricultural fertilizers. When it is not biologically removed from streams and rivers, excess, anthropogenically-derived nitrogen ends up in estuaries and coastal areas, where it is implicated in eutrophication, alteration of food webs, and hypoxia (Martinetto et al., 2006; Paerl et al., 2006; Diaz and Rosenberg, 2008). Nitrate can be lost from these systems via anaerobic ammonium oxidation (anammox) to dinitrogen gas or denitrification, i.e., the respiratory reduction of nitrate to either the potent greenhouse gas nitrous oxide or dinitrogen gas. Alternatively, it can be retained in the system as biologically available ammonium via dissimilatory nitrate reduction to ammonium (DNRA), with possible nitrogen losses via trace amounts of nitrous oxide (Smith, 1982; Cruz-García et al., 2007; Giblin et al., 2013). For a long time, it was thought that denitrification was the main nitrate/nitrite removing process in coastal ecosystems (Burgin and Hamilton, 2007), outcompeting anammox and DNRA in dynamic, eutrophic estuaries (Trimmer et al., 2003; Rich et al., 2008; Dale et al., 2009; Giblin et al., 2013). Since, the first reports on DNRA in estuarine environments approximately 40 years ago (Buresh and Patrick, 1978), an increasing number of studies indicated that DNRA is more relevant in nitrate/nitrite turnover in these systems than previously assumed (Gardner et al., 2006; Koop-Jakobsen and Giblin, 2010; Dong et al., 2011; Giblin et al., 2013). However, in contrast to denitrification, DNRA has been underexplored for the last decades and, despite some attempts (Bonin, 1996; Yoon et al., 2015b), the key organisms carrying out this process in marine and estuarine environments, their response to varying environmental conditions and how the DNRA process itself relates to other nitrogen removing processes remain largely unknown.

DNRA is a facultative, two-step anaerobic process involving nitrate reduction to nitrite followed by the 6-electron reduction of nitrite to ammonium (Einsle et al., 1999), of which two different modes of energy conservation have been described. The respiratory mode generates a proton motive force by electron transport from non-fermentable organic substrates to nitrite resulting in ATP production (Simon, 2002), while in the fermentative mode, nitrite is an electron-sink allowing re-oxidation of NADH with the generation of one extra ATP by substrate level phosphorylation for each acetate produced (Cole and Brown, 1980; Polcyn and Podeszwa, 2009). Respiratory DNRA can also contribute to chemolithoautotrophic growth when coupled to the oxidation of reduced inorganic sulfur forms (hydrogen sulfide, sulfide, or elemental sulfur; Dalsgaard and Bak, 1994; Brunet and Garciagil, 1996). Nitrite reduction to ammonium can be catalyzed by the cytoplasmic NADH-dependent nitrite reductase NirB or its to two-subunit variant NirBD (Harborne et al., 1992) and/or the periplasmic pentaheme cytochrome *c* nitrite reductase NrfA (Einsle et al., 1999), depending on the organism and growth conditions. *Escherichia coli* and *Bacillus vireti* were shown to harbor and express genes for both enzymes (Cole, 1996; Mania et al., 2014), while other DNRA organisms such as *Wollinella succinogenes* (Simon, 2002), *Bacillus subtilis* (Nakano and Zuber, 1998), and *Archaea* (Rusch, 2013) contain either *nrfA* or *nirB*. Furthermore, in *E*. *coli*, differential expression of *nrfA* and *nirB* under low and high nitrate concentrations respectively was observed (Wang and Gunsalus, 2000). DNRA-related ecophysiology, enzymology, gene expression and regulation have been extensively studied in model organisms like *E. coli, W. succinogenes*, and *B. subtilis* (Cole, 1996; Nakano and Zuber, 1998; Simon, 2002), and more recently also in *B. vireti* (Mania et al., 2014) and *Shewanella loihica* (Yoon et al., 2015a,b). Whole genome sequence analyses, however, demonstrated that DNRA, similar to denitrification, is phylogenetically very widespread, and can be found in members of *Bacteroidetes* (Mohan et al., 2004), *Proteobacteria* (*Gamma*-, *Delta*-, and *Epsilon*; Smith et al., 2007), *Actinobacteria, Firmicutes, Acidobacteria, Chloroflexi*, and *Planctomycetes* (Welsh et al., 2014). Some DNRA bacteria have furthermore been shown to contain partial or complete suites of genes for both DNRA and denitrification in their genome (Heylen and Keltjens, 2012; Sanford et al., 2012; Yoon et al., 2013; Mania et al., 2014). Functional capacity to carry out both nitrate reducing processes and the environmental drivers of nitrate partitioning to either process, like carbon-to-nitrogen ratios and nitrite concentrations, have thus far only been demonstrated for the marine strain *Shewanella loihica* PV-4 (Yoon et al., 2015a,b). In addition to pure culture experiments, natural prokaryotic communities have also been used for examining these environmental drivers (Kraft et al., 2014; Van Den Berg et al., 2015). To date, only limited cultured DNRA bacteria, including only few from marine environments, are available (Cole et al., 1974; Bonin, 1996; Hoffmann et al., 1998; Mania et al., 2014; Yoon et al., 2015b), and no recent attempts have been made to isolate new (marine) members.

The Westerschelde estuary is an eutrophied system characterized by a nitrogen load of 5 × 10^9^ mol N yr^−1^ (Soetaert and Herman, 1995) with nitrate being the predominant form of reactive nitrogen (Soetaert et al., 2006). Furthermore, denitrification and not DNRA was previously reported to be the predominant nitrate removing process (Dahnke et al., 2012; Van Colen et al., 2012). To better comprehend the relative importance of denitrification and DNRA in these estuarine sediments, nitrogen reduction potentials were determined of sediments obtained from the Paulina polder mudflat (Westerschelde estuary, SW Netherlands). In addition, to increase the knowledge on the organisms involved, nitrate reducing bacteria were isolated from enriched sediment cultures.

## Materials and methods

### Sampling

Sediment samples were collected at the Paulina polder mudflat (51° 21′ 24” N, 3° 42′ 51″ E) in collaboration with NIOZ, which provided the necessary permit for field sampling, issued by the “Provincie Zeeland, The Netherlands; Directie Ruimte, Milieu en Water.” They were taken using a plexiglas corer (Ø 6.2 cm). Samples for isolation of nitrate reducing bacteria were collected in October 2011 and samples for determination of the nitrogen reduction potential in October 2014. The latter samples were collected in triplicate at 5 different sampling sites over a distance of 1.6 m and stored at 4°C until further processing. Back in the lab, the upper cm of the sediment cores, containing the oxic-anoxic border (Van Colen et al., 2012; Decleyre et al., 2015) and main zone for dissimilatory nitrogen reduction was sampled. Triplicate samples were pooled per sampling site to include as much spatial variation as possible and subsequently stored in sterile falcon tubes at −80°C. Physico-chemical characteristics of samples collected in October 2014 were determined as described previously (Decleyre et al., 2015). Statistical differences in physicochemical parameters between all five sampling sites were evaluated using One-way ANOVA and *post hoc* tests in SPSS 21 (IBM SPSS Statistics for Windows, Version 21.0. Released 2012. Armonk, NY: IBM Corp.).

### Determination of nitrogen reduction potential

Nitrogen reduction potential was measured in triplicate using the acetylene inhibition technique according to Sørensen (Sørensen, 1978). Briefly, a 15 ml serum vial was filled with 2 g (ww) sediment (thawed at 37°C for 8 min) and 2 ml sterilized natural seawater (NSW). To prevent nitrogen limitation, the NSW was supplemented with 5 mM KNO_3_. No additional carbon source was added as preliminary experiments (data not shown) demonstrated that sediment/NSW contained sufficient carbon to support anaerobic respiration and/or fermentation. Synthesis of new enzymes was inhibited using 0.1 mM chloramphenicol allowing potential activity measurement of *in situ* expressed nitrogen reducing enzymes (Murray and Knowles, 1999). The vials were sealed with black butyl stoppers and aluminum crimps, and flushed five times with helium to remove oxygen. After adding 10% or 101.3 hPa acetylene, the vials were incubated in the dark at 15°C and at a constant stirring rate of 100 rpm. The nitrous oxide and carbon dioxide concentrations of all replicates were measured every hour (T_1_ to T_5_). Initial and final nitrite/ammonium concentrations were determined for each vial. Denitrification, DNRA, and nitrate to nitrite reduction rates were calculated using linear regressions (Table S1). No corrections were done for potential (i) overestimation of DNRA rates due to ammonium release by remineralization of organic matter during denitrification, and (ii) underestimation of denitrification rates due to incomplete inhibition of nitrous oxide reductase by acetylene (Groffman et al., 2006). Statistical differences in production rates between all five sampling sites were assessed using the non-parametric Kruskal–Wallis *H*-test in SPSS 21.

### Growth media

Growth conditions used in this study were defined by a set of variable and fixed parameters (Table 1) and growth media were prepared with sterile NSW collected form the Westerschelde estuary (Paulina polder) in an attempt to mimic natural conditions. They were based on the mineral medium of Stanier (Stanier et al., 1966) with slight modifications. Hepes (10 mM) was used as buffering agent, while phosphate was limited to 300 μM KH_2_PO_4_ based on the Redfield ratio (Redfield, 1934), to avoid decreased culturability as a consequence of high phosphate concentration (Bartscht et al., 1999). Iron, proven to be an essential element necessary for optimal growth of marine bacteria (D'Onofrio et al., 2010), was added as Fe(III)Na EDTA in a concentration (40 μM) mimicking the *in situ* concentrations found in the Westerschelde estuary (based on Schelde Monitoring database, http://www.scheldemonitor.be). Agarose (0.8%) was used as solidifying agent to eliminate potential growth inhibiting effects of agar (Tanaka et al., 2014). Other media components varied: signaling compound cyclic adenosine monophosphate (cAMP) at 0 or 10 μM; molar C/N ratio at 5 or 25, either with KNO_3_ or a combination of KNO_3_/KNO_2_ as nitrogen source (always with a total N concentration of 5 mM); glucose (designated as DNR2 media), a combination of sodium succinate dibasic hexahydrate/ethanol/glycerol (DNR3 media) or sodium pyruvate/ sodium acetate anhydrous (DNR4 media) as carbon source (Table 1). In addition, 10-fold diluted marine broth (MB) (BD Difco) (DNR1 media) supplemented with 5 mM of nitrate was also included as complex medium. Incubation temperature was set at 15°C as this approximates the yearly averaged temperature in the Westerschelde estuary. A detailed overview of all 26 growth media used in this study is given in Table S2.

**Table 1:** **Fixed and variable parameters of the growth conditions**.

**Fixed parameter**		**Variable parameters**	
Incubation temperature	15°C	Medium	1/10 MB
pH	7.2		Stanier mineral medium
Buffering agent	Hepes	C-sources	Glucose
NH^+^_4_ background concentration	4 mM		Succinate-ethanol- glycerol
Fe(III)Na EDTA	40 μM		Pyruvate-acetate
Vitamin solution	1 ml/L	N-sources	KNO_3_
Medium	NSW		KNO_3_/KNO_2_
N concentration	5 mM	C:N ratio (Molar C:N)	5 or 25
Atmosphere	anaerobic	Signaling factor	cAMP

### Enrichment, isolation, and cryopreservation of marine isolates

Enrichment cultures were set up in liquid medium under anaerobic, nitrate-reducing conditions. Sediment (1 g) was vortexed with 9 ml NSW for 15 min, and subsequently diluted 10-fold up to 10^−10^ in 120 ml serum vials for each growth medium. The vials were sealed with black butyl stoppers and aluminum crimps, and flushed five times with helium to remove oxygen (overpressure of 0.3 bar). For each dilution series, an additional vial was prepared without inoculum to check for potential nitrosation reactions in sterile medium (Mania et al., 2014). After adding 10% acetylene and 10% carbon dioxide to the headspace, the vials were incubated in the dark at 15°C and at a constant stirring rate of 100 rpm. Headspace concentrations of nitrous oxide and carbon dioxide were determined weekly.

The two highest dilutions of each growth medium producing nitrous oxide for two consecutive weeks were used for isolation, because (i) DNRA bacteria also produce nitrous oxide as a side product, (ii) denitrifying DNRA bacteria were not to be excluded and (iii) ammonium production as proxy for DNRA in enrichments is hampered by remineralization of organic matter. For each enrichment, dilutions were made in sterile NSW (10^−1^, 10^−2^, and 10^−3^ dilution, if necessary 10^−4^ and 10^−5^) and 100 μl of diluted culture was plated on solid media. Incubation was done at 15°C in an anaerobic gas container (BD Gaspack Container System) with an anaerobic indicator (Microbiology Anaerotest) and anaerobic BD Gaspack sachets. Colony formation was checked weekly. If no additional colonies were formed for two consecutive weeks, five isolates with different colony morphology were picked from each medium, and subsequently purified on identical solid medium. Finally, isolates were dereplicated based on their 16S rRNA gene identity and the type of medium they were isolated from, i.e., a representative of each group of highly related isolates was retained for further analyses.

All isolates were preserved at −80°C as described previously (Vekeman and Heylen, 2015). In short, isolates obtained from defined media were preserved in 10% DMSO prepared with NSW. For DNR1 and DNR2 type media, 1/10 MB or glucose (4.17 mM or 20.83 mM) was additionally added to the respective 10% DMSO-NSW solution as an extra cryoprotectant.

### Determination of dissimilatory nitrogen metabolism

To test whether the obtained isolates were strictly dependent on nitrate/nitrite as electron acceptor in the absence of oxygen or, alternatively, could use other non-defined electron acceptors present in NWS, media without added nitrogen were used. To determine the nitrogen reducing metabolism of each individual isolate, standardized growth experiments were performed in duplicate for each isolate with start- and endpoint determination of concentrations of nitrite, ammonium, nitrous oxide, and carbon dioxide. A 120 ml serum vial containing 19.8 ml liquid medium was inoculated with 200 μl cell suspension of OD 0.1 from each selected isolate (for slow growing isolates OD 0.05 was used). All isolates were tested in both complex (1/10 MB) and mineral media (for DNR1 isolates, DNR3 mineral media were used) to take into account the effect on our measurements of undefined N-compounds in marine broth. Blanks for each medium type were also included to detect potential nitrosation reactions (Mania et al., 2014). Positive controls for denitrification (*Paracoccus denitrificans* LMG 4049) and DNRA (*E. coli* LMG 5584) were included for all media. Incubation was performed at 15°C under anaerobic headspace with 10% acetylene and 10% carbon dioxide. Time of endpoint sampling was determined based on visual assessment of growth. An isolate was considered a denitrifier when 80% conversion of nitrate to nitrous oxide coincided with growth (Mahne and Tiedje, 1995) and a DNRA bacterium if the sum of nitrate reduction products (nitrite and nitrous oxide) was less than 70% of the consumed nitrate (Bonin, 1996) with concomitant ammonium production.

### Analytical methods

Nitrous oxide and carbon dioxide were detected and quantified using a Compact GC (Global Analyzer Solutions, Belgium) equipped with two columns (oxygen/nitrogen and carbon dioxide/nitrous oxide separation) connected to a thermal conductivity detector. The change in pressure due to nitrous oxide/carbon dioxide production was monitored with an infield seven pressure meter (UMS, Germany). Values obtained by gas chromatography were converted to μmol gas L^−1^_Liquid_ by compensating for change in gas pressure (measured with the Infield seven pressure meter) and taking the solubility of the gases into account. Samples for colorimetrics (500 μl of liquid culture) were pretreated using KCl to avoid inhibition of amines (Keeney and Nelson, 1987). Nitrite was determined using the Griess reaction (Griess, 1879) and ammonium using the salicylate-nitroprussidine method (Baethgen and Alley, 1989).

### 16S rRNA and *nrfA* gene sequence analyses

DNA was extracted from each isolate by the guanidium-thiocyanatelectronEDTA-sarkosyl method (Pitcher et al., 1989). Amplification and sequencing of the complete 16S rRNA gene was performed as described previously (Heyrman and Swings, 2001). Sequences were assembled using the BioNumerics 7.0 software (Applied Maths). Finally, the EzTaxon server [http://www.ezbiocloud.net/eztaxon; (Kim et al., 2012)] was used to taxonomically assign each isolate to a genus. Maximum likelihood analyses of 16S rRNA genes of the isolates obtained in this study together with previously identified DNRA bacteria (phenotypically characterized or based only on the presence of the *nrfA* gene) were performed to assess the diversity of DNRA bacteria obtained. Therefore, the *nrfA_*Welsh data set in the Fungene database containing *nrfA* sequences obtained from whole genomes was used to select representatives of each genus of the currently known taxonomic diversity (Fish et al., 2013). After checking the *nrfA* genes for the presence of the key KXRH or KXQH motifs and 5 heme groups—this to prevent inclusion of closely related octaheme nitrite reductase (ONR) or other multiheme cytochrome *c* proteins—the corresponding 16S rRNA gene sequence of each representative was obtained from the NCBI database for inclusion in the comparison. A profile-based multiple sequence alignment of the obtained 16S rRNA gene sequences was subsequently achieved using the SILVA Incremental Aligner (SINA v1.2.11; Pruesse et al., 2012). Maximum likelihood analysis was performed in RaxML 7.4.2 using a general time reversible model with gamma distributed rates (GTR+G; Stamatakis, 2006; Ott et al., 2010).

In addition, *nrfA* gene amplification was performed on all isolates using primer sets F1-7R1 (Mohan et al., 2004), F2-7R1 (Mohan et al., 2004), and nrfAF2aw-nrfAR1 (Welsh et al., 2014). To prevent interference of non-specific amplification during sequencing, amplicons obtained with Mohan primers (Mohan et al., 2004) were extracted from an agarose gel and subsequently used for sequencing. The *nrfA* identity of obtained amplicons was verified by checking for the presence of NrfA diagnostic motifs, i.e., KXRH or KXQH, as all three primer sets targeted the region between the third and the fourth heme binding motif (Mohan et al., 2004; Welsh et al., 2014).

### Nucleotide accession numbers

The nucleotide sequences of the 16S rRNA and *nrfA* data generated in this study have been deposited in the GenBank database under accession numbers KT185111-KT185193 and KT159169-KT159180 respectively.

## Results and discussion

### Nitrogen reduction potential of Westerschelde sediment at meter scale

**Figure 1:**
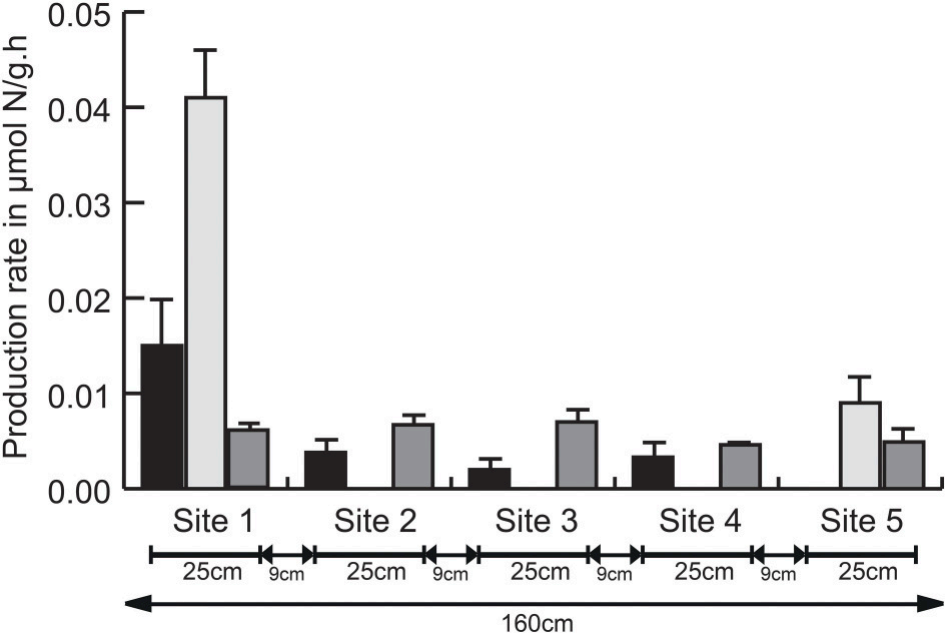
**Averaged nitrite, ammonium, and nitrous oxide production rates (± SD) per sampling site (***n*** = 3) over a period of 5 h (= T_1_–T_5_)**. For clarity, one sided error bars are shown. Black bars: nitrate reduction to nitrite, light gray bars: dissimilatory nitrate reduction to ammonium (DNRA), dark gray bars: denitrification. Total distance between all five sampling sites (1.60 m) and individual distance between all sampling sites is represented. Detailed information on the physico-chemical composition of these five sites is found in Table S3.

Despite their ecological importance, knowledge on DNRA processes in marine environments remains scarce. In the past, denitrification was considered the dominant marine nitrogen reduction pathway, while DNRA contributions were minimalized or even ignored (Burgin and Hamilton, 2007). Recent studies in marine and estuarine environments, however, have demonstrated that DNRA can also be the predominant nitrogen reduction pathway. Giblin et al. (2013) showed DNRA dominated total nitrogen reduction in approximately one-third of 55 coastal sediment sites examined. Similarly, Song et al. (2014) found benthic DNRA to be responsible for almost half of the nitrogen removal across the New River estuary, with DNRA rates exceeding those of denitrification (Lisa et al., 2014). Spatial variation in nitrogen removal rates is often assessed on a regional or local scale (Song et al., 2014; Smith et al., 2015), but not on meter or even smaller scale. Here, potential nitrogen removal rates, i.e., nitrate reduction to nitrite, denitrification (nitrous oxide measured with the acetylene method as a proxy) and DNRA, were measured across a 1.6 m scale, with five sampling sites approximately 9 cm from each other (Figure 1). Strikingly, we found significant differences in rate and dominance of the three processes at this small scale. Denitrification was observed at all sites, with significantly different rates (*p* < 0.05). In contrast, DNRA was limited to sites 1 and 5, located at a distance of 1.6 m, and appeared to be the dominant nitrogen removal process, with higher rates than denitrification (sites 1 and 5) and nitrate reduction to nitrite (site 1). Nitrite production was observed at all sites except site 5, with rates differing significantly between the five sampling sites (*p* < 0.05; Figure 1). The averaged nitrite, ammonium and nitrous oxide production rates were 0.0047 ± 0.0013 μmol N-NO^−^_2_/g.h, 0.01 ± 0.002 μmol N-NH^+^_4_/g.h, and 0.0058 ± 0.0003 μmol N-N_2_O/g.h respectively (individual rates in Table S4). Production rates of N-N_2_O observed in this study were not consistent with previous reports in marine sediments, they were either approximately one order of magnitude higher (Dul'Tseva et al., 2000; Magalhães et al., 2011) or nearly three orders of magnitude lower (Stock et al., 2014). Differences in the experimental set-up in these studies compared to ours, such as the non-inhibition of enzyme synthesis (Murray and Knowles, 1999) or the addition of extra carbon source, both leading to overestimation of denitrification rates (Bernot et al., 2003), are plausible explanations for the lower potential rates observed here. Furthermore, seasonal variability in time of sampling might also contribute to these observed differences. Nevertheless, potential rates of DNRA observed in sampling site 5 agreed with previous observations based on isotopic labeling experiments in estuarine sediments, while those of sampling site 1 were approximately 2-fold higher (Kelly-Gerreyn et al., 2001; An and Gardner, 2002; Song et al., 2014).

In addition to significantly different rates between the five sites for all three nitrogen removal processes, their occurrence was also site-dependent. Carbon to nitrogen ratio has long been considered the determining factor for nitrate partitioning to either DNRA or denitrification (Tiedje, 1988), which was recently confirmed in long-term incubations of marine sediments (Kraft et al., 2014) as well as chemostat experiment with *S*. *loihica*, a gammaproteobacterium containing the gene inventory for both DNRA and denitrification (Yoon et al., 2015b). Based on the significant differences in nitrate concentration in pore water and similar total organic carbon content (Table S3), denitrification is expected to be favored at sites 1 and 2 (low C:N ratio) while DNRA would dominate the three other sites. In contrast, DNRA was limited to sites 1 and 5, with low and high C:N ratio respectively and denitrification contributed to nitrogen removal at all sites. While nitrate sufficiency (bulk addition of 5 mM at the start of the experiment) may explain the overall occurrence of denitrification, C:N ratio was clearly not the main driver differentiating between both processes. Previous reports on the effect of pH on DNRA and denitrification were inconclusive (Stevens et al., 1998; Rutting et al., 2011), although DNRA was favored over denitrification at elevated pH in *S*. *loihica* (Yoon et al., 2015b). We did not monitor the pH on-site, but know from previous work that pH can be variable at the meter scale in the Paulina tidal flat (Decleyre et al., 2015). Additional key environmental controls that could explain the observed small scale variation were either not relevant, such as microbial generation time (Kraft et al., 2014) and supply of nitrite relative to nitrate (Kraft et al., 2014; Yoon et al., 2015a), or undetermined, such as the presence of free sulfides (hydrogen sulfide, sulfide) or elemental sulfur (Burgin and Hamilton, 2007). Site 1 with the highest DNRA rate did, however, contain the highest chl *a* concentration (Table S3), i.e., proxy for diatom biomass, although it was not significantly different from the four other sites. Nevertheless, it is plausible that diatoms, which are known to store nitrate intracellularly and us it as a dark survival strategy (Kamp et al., 2011), contribute to the high DNRA rate at that site. In addition, higher trophic levels like meiofauna can influence removal processes and rates but are rarely considered (Stock et al., 2014; here for example because of limited sample size). Nevertheless, the presence of meiofauna can directly (Frangoulis et al., 2005) or indirectly (Nascimento et al., 2012) increase organic matter, with subsequent stimulation of sulfate reduction (Berner and Westrich, 1985) resulting in hydrogen sulfide production, known to favor DNRA and autotrophic denitrification (Burgin and Hamilton, 2007; Moraes et al., 2012). Taken together, we expect that the combination of complex interactions between different trophic levels, the resulting microscale physico-chemistry and the highly dynamic nature of intertidal sediments might contribute to the observed significant variation in nitrogen removing processes at the meter scale.

### Marine dissimilatory reducers of nitrate to ammonium

With the advance of whole genome sequencing, the ability to carry out DNRA was shown to be phylogenetically more widespread than originally thought. Still, key organisms involved in DNRA in marine ecosystems and their ecophysiology remain largely unknown. Macfarlane and colleagues were the first to report DNRA capabilities of a *Vibrio* sp. and a *Clostridium butyricum* strain obtained from estuarine sediments (Keith et al., 1982; Macfarlane and Herbert, 1982). Years later, Bonin (1996) confirmed the DNRA capability of two other Gammaproteobacterial strains isolated from estuarine sediments and reported that nitrate limitation (1 mM) resulted in ammonium production while high nitrate levels (10 mM) caused nitrite accumulation and less efficient ammonium production. Recent studies on the marine strain *S. loihica* PV-4 that is able to perform both denitrification and DNRA, indicated that nitrate limitation (high C:N), high nitrite-to-nitrate ratio, alkaline pH and high temperatures favor DNRA over denitrification activity (Yoon et al., 2015a,b). These Gammaproteobacterial strains form the basis for our current knowledge of DNRA in marine ecosystems, yet their limited number underline the urgent need for new cultured marine representatives to further explore the ecophysiology of phylogenetically distinct DNRA organisms. Therefore, we enriched estuarine sediments under anaerobic, nitrate reducing conditions using 26 different growth media (under 10% acetylene), mimicking *in situ* physico-chemical conditions, and subsequently performed isolations. In total, 83 isolates, belonging to 27 genera of *Actinobacteria, Firmicutes, Bacteroidetes, Alpha*,- and *Gammaproteobacteria* (Table S5) were obtained. This partially agreed with available 16S rRNA pyrosequencing data from the same site (Decleyre et al., 2015), in which *Gammaproteobacteria* and *Bacteroidetes* were found to be most dominant. Although we did not apply an exhaustive isolation approach and only picked up five isolates per medium after elective enrichment, it is striking that only four of the 27 genera (*Martellela, Pseudoruegeria, Roseovarius*, and *Vibrio*) were found via both isolation and pyrosequencing. As for denitrifiers (Heylen et al., 2006), elaborate medium optimization is necessary to increase cultivated representatives for DNRA bacteria. Nevertheless, our study clearly showed that either diluted complex medium (representatives of 8 genera) or combined non-fermentable carbon sources (9 genera) are preferred over glucose (2 genera) (Table S5). Furthermore, addition of KNO_3_ as electron acceptor yielded twice as much diverse isolates compared to media supplemented with KNO_3_/KNO_2_ as nitrogen source (18 vs. 9 genera), the latter probably caused by organism-dependent nitrite intolerance (Table S5). Nevertheless, inclusion of nitrite (at low concentrations, i.e., 2 mM in this study) as electron acceptor is necessary to target bacteria lacking the genes for nitrate reductase but capable of nitrite reduction to ammonium or denitrification. *Paraoerskovia, Citrobacter, Shigella*, and *Halomonas* were only isolated from media containing both KNO_3_ and KNO_2_ (Table S5). None of the isolates appeared solely dependent on nitrate or nitrite as electron acceptor in the absence of oxygen. Growth was still observed without added electron acceptors suggesting that natural seawater, used to prepare the growth media to mimic *in situ* physico-chemical conditions, provided all isolates with alternative electron acceptors (e.g., manganese, iron, sulfate) to support growth. This made it impossible for us to recognize dissimilatory nitrogen reducers, i.e., isolates that are capable of nitrate or nitrite reduction to ammonium or dinitrogen, based solely on growth in nitrogen oxide amended media.

Therefore, unique representatives of each closely related group of isolates were selected based on their 16S rRNA gene identity and isolation medium, yielding 35 isolates for detailed determination of their dissimilatory nitrogen metabolism. In batch experiments, isolates were grown in their isolation medium and 10-fold diluted marine broth supplemented with 5 mM KNO_3_ or KNO_3_/KNO_2_ (depending on original isolation conditions). Concentrations of potential end-products nitrite, ammonium and nitrous oxide were determined at end-point. Fifteen out of the 35 isolates were shown to actually reduce nitrate as electron acceptor. No denitrifying bacteria were isolated [confirmed by negative results of *nirK* and *nirS* PCR (data not shown)], but rather all isolates had a DNRA phenotype, capable of producing ammonium from nitrate. For 12 out of 15 nitrate reducers, the DNRA phenotype was re-confirmed with the detection and sequencing of the *nrfA* gene (Table 2 and Figure S1). The three remaining isolates either contained divergent *nrfA* genes not targeted by the primers used or harbored *nirB*. The *nirB* gene is unfortunately a poor marker gene because of its role in both assimilatory and dissimilatory nitrate reduction to ammonium and general primers are currently lacking. The lack of denitrifiers among the isolates was initially surprising, as growth media were nitrate sufficient (>1 mM) and nitrous oxide producing dilutions were selected for isolation (note that nitrous oxide production was used as a selection criterion because DNRA bacteria also produce nitrous oxide as side product, denitrifying DNRA bacteria were not to be excluded and ammonium production as proxy for DNRA in enrichments is hampered by remineralization of organic matter). When looking at the data in more detail however, this makes sense. The amount of nitrous oxide produced by the enrichment cultures ranged from 0.09 to 0.6 mM, i.e., between 3.6 and 24% of all nitrate was converted to nitrous oxide. This is higher than one would expect from a pure culture DNRA bacterium (from 0.1 to 5%, depending on the organism; Streminska et al., 2012), but much lower than expected for a denitrifier (80–100%; Mahne and Tiedje, 1995). So, this range of nitrous oxide production from the enrichment cultures suggested a mix of denitrifiers and DNRA bacteria. The exclusive isolation of DNRA bacteria might point toward numerical dominance of DNRA bacteria in the enrichments, but this was not verified in additional tests.

**Table 2:** **Identification of cultured nitrate/nitrite ammonifiers retrieved from estuarine sediments**.

**Taxonomy**	**Isolate number**	**Type strain with the highest 16S rRNA gene sequence similarity to query sequences**			**Nitrate metabolism**				***nrfA*** **gene amplification**		
		**Species name**	**% sim**	**Accession number**	**NO^−^_3_ reduction to NO^−^_2_**	**Denitrification**	**DNRA**	**N_2_O production (%)^a^**	**F1-7R1^b^**	**F2-7R1^b^**	**F2aw-7R1^c^**
*Alphaproteobacteria*											
*Rhodobacterales*	R-52651	*Celeribacter baekdonensis* L-6^T^	100	HM997022	+	−	+	0–1.1	−	+	−
*Rhodospirillales*	R-52913	*Thalassospira lucentensis* DSM 14000^T^	99.6	AM294944	+	−	+	0–0.3	−	−	−
	R-52699		99.6	AM294944	+	−	+	0–0.7	−	−	−
*Gammaproteobacteria*											
*Aeromonadales*	R-52674	*Oceanisphaera donghaensis* BL1^T^	99.77	DQ190441	+	−	+	2–2.1	+	+	−
*Alteromonadales*	R-52649	*Shewanella colwelliana* ATCC 39565^T^	100	AY653177	+	−	+	0.3–1.3	−	+	−
	R-52673	*Shewanella marisflavi* SW 117^T^	100	AY485224	+	−	+	0.8–1.4	+	+	−
*Enterobacteriales*	R-52910	*Citrobacter gillenii* CDC 4693-86^T^	99.9	AF025367	+	−	+	0.6–2.9	+	+	+
*Oceanospirillales*	R-52914	*Halomonas denitrificans* M29^T^	98.9	AM229317	+	−	+	2.4–2.9	+	+	+
*Vibrionales*	R-52677	*Vibrio alginolyticus* NBRC 15630^T^	99.7	CP006718	+	−	+	0.8–2	+	+	+
	R-52915		99.4	CP006718	+	−	+	0.9–2.1	+	+	+
	R-52696		99.4	CP006718	+	−	+	1.2–1.7	+	+	+
	R-52683	*Vibrio diabolicus* HE800^T^	99.4	X99762	+	−	+	1–1.9	−	+	−
	R-52669	*Vibrio neocaledonicus* NC470^T^	99.4	JQ934828	+	−	+	0.7–2.4	−	−	−
	R-52688		99.79	JQ934828	+	−	+	0.7–2.4	+	+	+
	R-66650	*Vibrio rumoiensis* S-1^T^	100	AB013297	+	−	+	0.3–2	+	+	−

*Taxonomic assignment to genus level based on the 16S rRNA gene sequence analysis, observed dissimilatory reduction of nitrogenous compounds, amounts of nitrous oxide produced, and nrfA amplification results are represented. Reduction of nitrate to nitrite and DNRA have been tested in two different growth conditions (complex or mineral medium)*.

a *Percentage of trace amounts of nitrous oxide detected in both 1/10 marine both and mineral media supplemented with 5 mM nitrate*.

b *nrfA gene amplification primers (505 bp and 231 bp amplicon respectively) from Mohan et al. (2004)*.

c *nrfA gene amplification primers (269 bp amplicon) from Welsh et al. (2014)*.

Our data reconfirms the ability of members of *Vibrio* (Liu et al., 1988), *Shewanella* (Yoon et al., 2015b), and *Citrobacter* (Smith, 1982) to perform DNRA, while demonstrating for the first time this capability for members of *Halomonas, Thalassospira, and Celeribacter*, previously only reported to perform nitrate reduction and/or (partial) denitrification (Peyton et al., 2001; Liu et al., 2007; González-Domenech et al., 2010). Llamas and colleagues suspected DNRA in *Halomonas maura* (Llamas et al., 2006), but did not test it physiologically. *NrfA* amplicons were obtained from *Halomonas* sp. R-52914 and in *Celeribacter* sp. R-52651, while this was not the case for *Thalassospira* sp. R-52913 and R-52699. *In silico* analysis of all six publically available genome sequences of *Thalassospira* strains revealed *nirB* genes instead of *nrfA*, which might explain why all three *nrfA* primer sets failed to render an amplicon. This might also be the case for *Vibrio* sp. R-52669, although the closely related R-52688 did render a *nrfA* amplicon. Still, strain-dependent differences in dissimilatory nitrogen reduction geno- and phenotype are not uncommon (Kloos et al., 2001; Falk et al., 2010; Liu et al., 2013).

**Figure 2:**
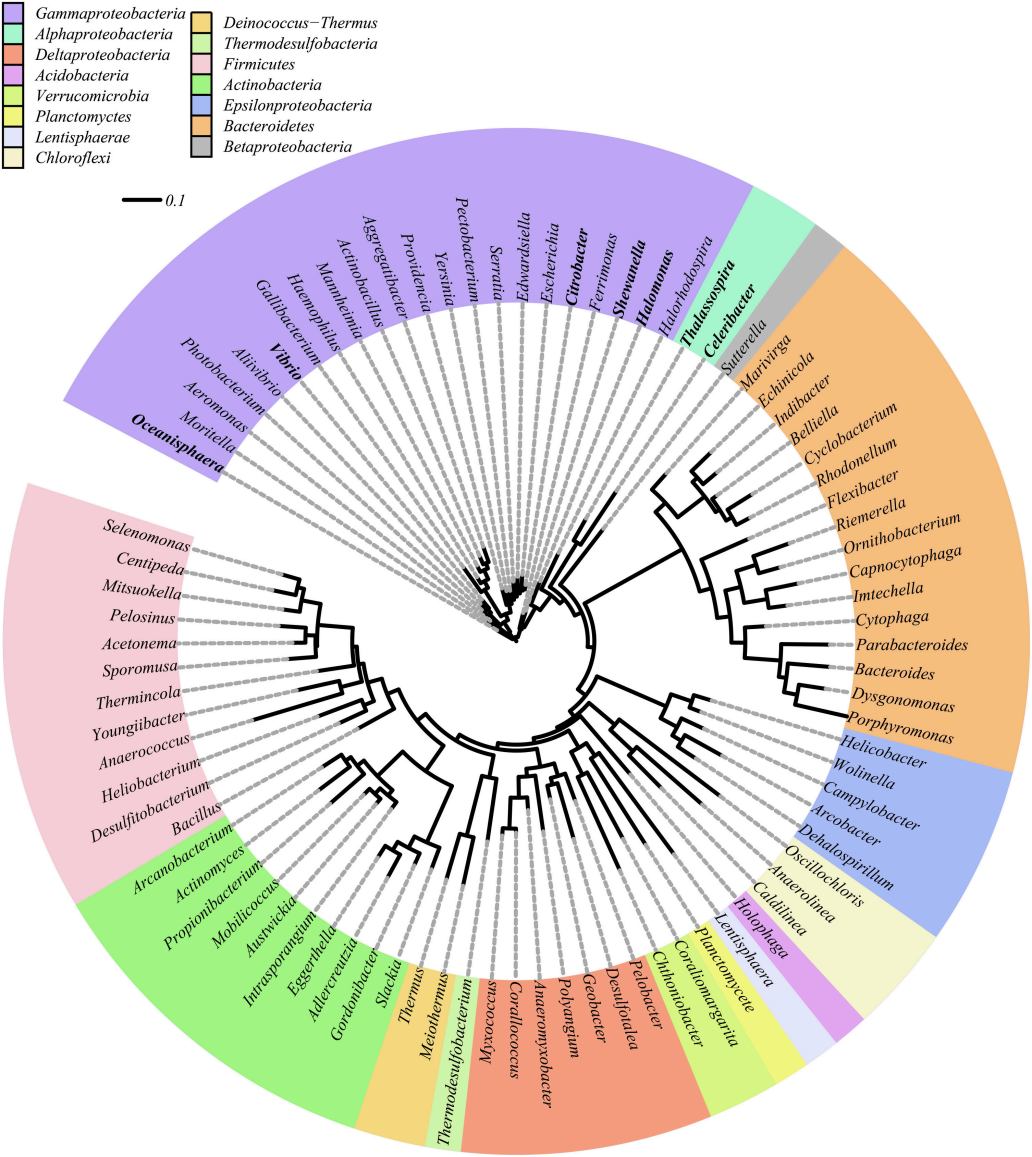
**Maximum likelihood phylogenetic analysis of 16S rRNA genes of previously known DNRA bacteria as determined by the presence of a ***nrfA*** gene**. Genera found during this study are indicated in bold.

All DNRA isolates obtained in this study belonged to the *Gammaproteobacteria* and *Alphaproteobacteria* (Table 2). An overview of diverse phyla reported to harbor DNRA bacteria, either tested phenotypically or by *nrfA* gene amplification, can be found in Figure 2. In contrast to previous reports of DNRA phenotype predominantly being found in *Gammaproteobacteria* (Forsythe et al., 1988; Liu et al., 1988; Bonin, 1996; Yoon et al., 2015b), here a wide variety was found of phylogenetically unrelated microorganisms belonging to 11 different phyla harboring the potential to perform DNRA. Such a broad taxonomic distribution was also previously observed for denitrifying organisms (Philippot, 2002). Furthermore, the observed diversity contrasts enormously with the number of physiologically tested representatives, i.e., limited to *Gammaproteobacteria* (Smith, 1982; Keith and Herbert, 1983; Liu et al., 1988; Yoon et al., 2015b), *Firmicutes* (Keith et al., 1982; Hoffmann et al., 1998; Mania et al., 2014) and *Alphaproteobacteria* (this study), underlining the previous underestimation of DNRA organism diversity.

## Conclusion

Denitrification in marine environments is generally accepted to contribute substantially to nitrogen removal. Reports on the comparable or higher contribution of DNRA to nitrogen removal have revived the scientific interest in DNRA, physico-chemical parameters determining nitrate partitioning to denitrification and DNRA, the relative importance of the key players *in situ* and their ecophysiology. Here, we demonstrate that small scale heterogeneity in intertidal sediments influences the occurrence and rates of dissimilatory nitrogen reduction processes. Whereas, denitrification rates were comparable at the cm to m scale, DNRA and nitrate reduction to nitrite was site-specific and could vary significantly within 25 cm. Key environmental drivers partitioning nitrate among these processes could not be identified but did not relate to carbon to nitrogen ratio. Furthermore, 15 DNRA strains were obtained from estuarine sediments, including members of *Thalassospira, Celeribacter*, and *Halomonas* previously unrecognized DNRA organisms. These novel environmental strains are now available for further ecophysiological studies on DNRA.

### Conflict of interest statement

The authors declare that the research was conducted in the absence of any commercial or financial relationships that could be construed as a potential conflict of interest.
