# Supplementary material for: Dissimilatory nitrogen reduction in intertidal sediments of a temperate estuary: small scale heterogeneity and novel nitrate-to-ammonium reducers
Source: Front Microbiol. 2015 Oct 14;6:1124. doi: 10.3389/fmicb.2015.01124 (PMC4604302; doi:10.3389/fmicb.2015.01124)
Supplement: Supplementary file 1 [file DataSheet1.DOCX]

***Supplementary Material***

# Dissimilatory nitrogen reduction in intertidal sediments of a temperate estuary: small scale heterogeneity and novel nitrate to ammonium reducers.

# Decleyre Helen^1^, Heylen Kim^1,*^, Van Colen Carl^2^, Willems Anne^1^

**^*^Correspondence:** Dr. Kim Heylen, Laboratory of Microbiology (LM-UGent), Department of Biochemistry and Microbiology, Ghent University, K.L. Ledeganckstraat 35, 9000 Ghent, Belgium,
[Kim.Heylen@UGent.be](mailto:Kim.Heylen@UGent.be)

**1.1 Supplementary figures**

R-52649 ---------- ---------- ---------- ---------- ---------- ----------

R-52651 ---------- ---------- ---------- ---------- ---------- ----------

R-52677 ---------- ---------- ---------- ---------- ---------- ----------

R-52673 ---------- ---------- ---------- ---------- ---------- ----------

R-52674 ---------- ---------- ---------- ---------- ---------- ----------

R-52910 ---------- ---------- ---------- ---------- ---------- ----------

R-52683 ---------- ---------- ---------- ---------- ---------- ----------

R-52914 ---------- ---------- ---------- ---------- ---------- ----------

R-52915 ---------- ---------- ---------- ---------- ---------- ----------

R-66650 ---------- ---------- ---------- ---------- ---------- ----------

R-52696 ---------- ---------- ---------- ---------- ---------- ----------

R-52688 EVVNPIGCSD CHDTRSEKFN QGEPEVALTR PYVERAFDVI GKNFDEQSRL DKQASVCAQC

R-52649 ----YYFEK TKEKKGFVKFP WDMGTTVEQM EVYYDNMEFA DWTHAVSKTP MLKAQHPGYE

R-52651 ----YFNKNR VKGSAFVQLP WDKGMGVEEM EEYYDEMDFK DWTHKLSKAP MLKAQHPGYE

R-52677 --EYYFTGPT KA----VKFP WDMGTNVADM EKYYDALNFK DWTHAVSKAP MLKAQHPGFE

R-52673 ---------- ---------P WDMGTNVADM EKYYDALNFK DWTHAVSKAP MLKAQHPGFE

R-52674 ----YFTGPT KA----VKFP WDMGTTVGDM EKYYDALDFK DWTHAVSKAP MLKAQHPGFE

R-52910 ---YYFDGKN KA----VKFP WDEGMKVENM EKYYDAIAFS DWTNSLSKTP MLKAQHPEYE

R-52683 ---YYFDGKN KA----VKFP WDDGMKVENM EQYYDKIAFS DWTNSLSKTP MLKAQHPEYE

R-52914 --EYYFTGPT KA----VKFP WDMGTNVADM EKYYDALNFK DWTHAVSKAP MLKAQHPGFE

R-52915 ---YYFTGPT KA----VKFP WDMGTNVADM EKYYDALNFK DWTHAVSKAP MLKAQHPGFE

R-66650 ----YFTGPT KA----VKFP WDMGTNVADM EKYYDALNFK DWTHAVSKAP MLKAQHPGFE

R-52696 -------EKT KDRKGFVKFP WDMGTTVEQM EVYYDNMEFA DWTHALSKTP MLKAQHPGYE

R-52688 HVEYYFTGPT KA----VKFP WDMGTTVGDM EKYYDALDFK DWTHAVSKAP MLKAQHPGFE

R-52649 TWQLGVHGKN NVSCTDC--

R-52651 VYLKGIHADR GVSC-----

R-52677 TWRAGIHGKS KVVCVD---

R-52673 TWRAGIHGKN KVVCVDCHM

R-52674 TWREGIHGKN ---------

R-52910 TWTAGIHGKN NVTCID---

R-52683 ---------- ---------

R-52914 TWRAGIHGKN KVVCVD---

R-52915 TWRAGIHGKN K--------

R-66650 TWRAGIHGKN KVVCVDCHM

R-52696 TWQLGVHGKN NVSCTDC--

R-52688 TWREGIHGKN KVVCV----

**Fig. S1** Multiple sequence alignment of translated *nrfA* genes from twelve out of the fifteen isolates obtained in this study. Three different primer pairs were used, each targeting the region between the third and the fourth heme binding motif. NrfA identity of obtained amplicons was verified by the presence of a NrfA diagnostic motif (marked in yellow), *i*.*e*. KXRH or KXQH. In some cases, heme binding motifs were also detected as indicated in green.

**1.2 Supplementary tables**

Table S1 Time series data of sampling site 1. Individual rates per replicate, the standard error, the R² obtained after regression analysis and individual measurements of nitrite, ammonium and nitrous oxide concentrations per vial per replicate for each hour are represented.

|  | Replicate no. | Individual rates | SE | R² | Time serie data | |
| --- | --- | --- | --- | --- | --- | --- |
|  |  | (µmol N/ g.h) | (µmol N/ g.h) |  | h | (µmol N/ vial) |
| NO_2_^-^ | Rep1 | 1.64E-02 | 4.84E-03 | 0.66 | 1 | 0.00E+00 |
|  |  |  |  |  | 2 | 4.25E-03 |
|  |  |  |  |  | 3 | 4.09E-02 |
|  |  |  |  |  | 4 | 1.22E-01 |
|  |  |  |  |  | 5 | 2.35E-01 |
|  | Rep2 | 9.05E-03 |  | 0.64 | 1 | 9.21E-05 |
|  |  |  |  |  | 2 | 7.89E-03 |
|  |  |  |  |  | 3 | 2.00E-02 |
|  |  |  |  |  | 4 | 6.15E-02 |
|  |  |  |  |  | 5 | 1.34E-01 |
|  | Rep3 | 1.82E-02 |  | 0.74 | 1 | 0.00E+00 |
|  |  |  |  |  | 2 | 2.08E-02 |
|  |  |  |  |  | 3 | 6.83E-02 |
|  |  |  |  |  | 4 | 1.30E-01 |
|  |  |  |  |  | 5 | 2.47E-01 |
| NH_4_^+^ | Rep1 | 4.21E-02 | 4.59E-03 | 0.90 | 1 | 1.07E-01 |
|  |  |  |  |  | 2 | 1.08E-01 |
|  |  |  |  |  | 3 | 2.60E-01 |
|  |  |  |  |  | 4 | 2.86E-01 |
|  |  |  |  |  | 5 | 4.71E-01 |
|  | Rep2 | 4.48E-02 |  | 0.88 | 1 | 9.84E-02 |
|  |  |  |  |  | 2 | 2.40E-01 |
|  |  |  |  |  | 3 | 2.51E-01 |
|  |  |  |  |  | 4 | 2.91E-01 |
|  |  |  |  |  | 5 | 4.81E-01 |
|  | Rep3 | 3.58E-02 |  | 0.61 | 1 | 1.49E-01 |
|  |  |  |  |  | 2 | 1.88E-01 |
|  |  |  |  |  | 3 | 2.11E-01 |
|  |  |  |  |  | 4 | 2.53E-01 |
|  |  |  |  |  | 5 | 3.49E-01 |
| N_2_O | Rep1 | 6.30E-03 | 1.89E-04 | 0.93 | 1 | 7.65E-03 |
|  |  |  |  |  | 2 | 2.19E-02 |
|  |  |  |  |  | 3 | 3.06E-02 |
|  |  |  |  |  | 4 | 5.44E-02 |
|  |  |  |  |  | 5 | 5.35E-02 |
|  | Rep2 | 6.00E-03 |  | 0.94 | 1 | 3.41E-03 |
|  |  |  |  |  | 2 | 2.05E-02 |
|  |  |  |  |  | 3 | 3.26E-02 |
|  |  |  |  |  | 4 | 5.28E-02 |
|  |  |  |  |  | 5 | 6.11E-02 |
|  | Rep3 | 5.95E-03 |  | 0.94 | 1 | 6.45E-03 |
|  |  |  |  |  | 2 | 1.75E-02 |
|  |  |  |  |  | 3 | 3.27E-02 |
|  |  |  |  |  | 4 | 5.29E-02 |
|  |  |  |  |  | 5 | 5.97E-02 |

Table S2 Overview of variable parameters between all various growth media tested.

| Medium | Type of medium | | C-source | | | N-sources | | C:N ratio (molar) | | cAMP ^(a)^ |
| --- | --- | --- | --- | --- | --- | --- | --- | --- | --- | --- |
|  | Complex | Mineral | Glucose (mM) | Succinate; ethanol; glycerol (mM) | pyruvate; acetate (mM) | NO_3_^-^ (mM) | NO_3_^-^/NO_2_^-^ (mM) | Low (5) | High (25) |  |
| DNR1P1 | x |  |  |  |  | 5 |  |  |  | x |
| DNR1P2 | x |  |  |  |  | 5 |  |  |  |  |
| DNR2P1 |  | x | 4.17 |  |  | 5 |  | x |  |  |
| DNR2P2 |  | x | 4.17 |  |  |  | 3/2 | x |  |  |
| DNR2P3 |  | x | 20.83 |  |  | 5 |  |  | x |  |
| DNR2P4 |  | x | 20.83 |  |  |  | 3/2 |  | x |  |
| DNR2P5 |  | x | 4.17 |  |  | 5 |  | x |  | x |
| DNR2P6 |  | x | 4.17 |  |  |  | 3/2 | x |  | x |
| DNR2P7 |  | x | 20.83 |  |  | 5 |  |  | x | x |
| DNR2P8 |  | x | 20.83 |  |  |  | 3/2 |  | x | x |
| DNR3P1 |  | x |  | 2.08; 4.17; 2.78 |  | 5 |  | x |  |  |
| DNR3P2 |  | x |  | 2.08; 4.17; 2.78 |  |  | 3/2 | x |  |  |
| DNR3P3 |  | x |  | 10.42; 20.84; 13.89 |  | 5 |  |  | x |  |
| DNR3P4 |  | x |  | 10.42; 20.84; 13.89 |  |  | 3/2 |  | x |  |
| DNR3P5 |  | x |  | 2.08; 4.17; 2.78 |  | 5 |  | x |  | x |
| DNR3P6 |  | x |  | 2.08; 4.17; 2.78 |  |  | 3/2 | x |  | x |
| DNR3P7 |  | x |  | 10.42; 20.84; 13.89 |  | 5 |  |  | x | x |
| DNR3P8 |  | x |  | 10.42; 20.84; 13.89 |  |  | 3/2 |  | x | x |
| DNR4P1 |  | x |  |  | 4.17; 6.25 | 5 |  | x |  |  |
| DNR4P2 |  | x |  |  | 4.17; 6.25 |  | 3/2 | x |  |  |
| DNR4P3 |  | x |  |  | 20.83; 31.25 | 5 |  |  | x |  |
| DNR4P4 |  | x |  |  | 20;83; 31;25 |  | 3/2 |  | x |  |
| DNR4P5 |  | x |  |  | 4.17; 6.25 | 5 |  | x |  | x |
| DNR4P6 |  | x |  |  | 4.17; 6.25 |  | 3/2 | x |  | x |
| DNR4P7 |  | x |  |  | 20.83; 31.25 | 5 |  |  | x | x |
| DNR4P8 |  | x |  |  | 20.83; 31.25 |  | 3/2 |  | x | x |
| ^(a)^    Cyclic Adenosine MonoPhosphate | | | |  |  |  |  |  |  |  |

Table S3 Physico-chemical parameters of the five sampling sites used for determination of denitrification potential (n=3). Significant differences in pore water nitrate concentration between site 1,2 (indicated by ^*^) and 3,4,5 (indicated by ^+^) could be detected (p < 0.05). No significant differences between site 1 and 2 or sites 3,4 and 5 could be detected. All other parameters were found to be not significantly different between all five sampling sites.

| Parameter | Sampling site 1 | Sampling site 2 | Sampling site 3 | Sampling site 4 | Sampling site 5 |
| --- | --- | --- | --- | --- | --- |
| TOM (%)^a^ | 3.87 ± 0.07 | 3.26 ± 0.20 | 3.65 ± 0.31 | 3.79 ± 0.08 | 3.58 ± 0.40 |
| [Chl *a*] (µg/g dw) | 27.19 ± 4.51 | 19.03 ± 4.93 | 17.97 ± 3.40 | 19.05 ± 2.47 | 19.94 ± 5.15 |
| [EPS] (µg/mg dry sediment)^b^ | 0.28 ± 0.03 | 0.26 ± 0.05 | 0.27 ± 0.03 | 0.24 ± 0.01 | 0.26 ± 0.02 |
| % mud ^c^ | 47.59 ± 2.24 | 51.41 ± 2.72 | 47.74 ± 3.16 | 48.39 ± 1.00 | 49.18 ± 2.73 |
| [NH_4_^+^] (mg/l)^d^ | 2.76 ± 0.46 | 3.10 ± 0.52 | 3.71 ± 0.45 | 3.57 ± 0.48 | 3.51 ± 0.22 |
| [NO_3_^-^] (µg/l)^d^ | 220.04 ± 16.43^*^ | 207.56 ±65.31^*^ | 30.42 ± 22.44^+^ | 44.82 ± 7.01^+^ | 47.55 ± 37.84^+^ |
| [NO_2_^-^] (µg/l)^d^ | 8.48 ± 1.59 | 11.70 ± 4.06 | 6.35 ± 1.72 | 6.96 ± 1.01 | 5.62 ± 0.55 |
| [PO_4_^3-^] (mg/l)^d^ | 1.18 ± 0.15 | 1.14 ± 0.26 | 1.39 ± 0.34 | 1.42 ± 0.47 | 1.12 ± 0.07 |
| [Si] (mg/l)^d^ | 4.23 ± 1.36 | 4.63 ± 0.23 | 6.53 ± 0.83 | 5.79 ± 1.21 | 5.95 ± 0.92 |

a. TOM, total organic matter.
b. Extracellular polymeric substances.
c. Percentage mud (particle size < 63µm) determined using the Wenthworth grain size chart .
d. Pore water concentrations.

Table S4 Individual rates of nitrite, ammonium and nitrous oxide production per sampling site per replicate and the standard error.

| Replicate n° | Individual rates (µmol/g.h) | | | | | |
| --- | --- | --- | --- | --- | --- | --- |
|  | NO_2_^-^ | SE | NH_4_^+^ | SE | N_2_O | SE |
| 1.1 | 1.64E-02 | 4.84E-03 | 4.21E-02 | 4.59E-03 | 6.30E-03 | 1.89E-04 |
| 1.2 | 9.05E-03 |  | 4.48E-02 |  | 6.00E-03 |  |
| 1.3 | 1.82E-02 |  | 3.58E-02 |  | 5.95E-03 |  |
| 2.1 | 3.85E-03 | 8.13E-04 | 0.00E+00 | 0.00E+00 | 6.45E-03 | 3.04E-04 |
| 2.2 | 2.80E-03 |  | 0.00E+00 |  | 6.95E-03 |  |
| 2.3 | 4.40E-03 |  | 0.00E+00 |  | 6.40E-03 |  |
| 3.1 | 2.25E-03 | 3.28E-04 | 0.00E+00 | 0.00E+00 | 6.40E-03 | 4.65E-04 |
| 3.2 | 2.00E-03 |  | 0.00E+00 |  | 7.15E-03 |  |
| 3.3 | 2.65E-03 |  | 0.00E+00 |  | 7.25E-03 |  |
| 4.1 | 2.55E-03 | 4.31E-04 | 0.00E+00 | 0.00E+00 | 4.70E-03 | 7.64E-05 |
| 4.2 | 3.40E-03 |  | 0.00E+00 |  | 4.55E-03 |  |
| 4.3 | 3.10E-03 |  | 0.00E+00 |  | 4.65E-03 |  |
| 5.1 | 0.00E+00 | 0.00E+00 | 6.25E-03 | 3.82E-03 | 4.95E-03 | 5.77E-04 |
| 5.2 | 0.00E+00 |  | 7.35E-03 |  | 4.30E-03 |  |
| 5.3 | 0.00E+00 |  | 1.34E-02 |  | 5.45E-03 |  |

Table S5 Diversity of isolates retrieved from estuarine sediments. Taxonomic assignment to genus level based on the 16S rRNA gene sequence analysis, isolation conditions, number of isolates obtained per genus per type of medium and strain numbers are represented.

| Taxonomy | | Type strain with the highest 16S rRNA gene sequence similarity to query sequences | | | Isolation conditions | | No isolates | Isolate strain number |
| --- | --- | --- | --- | --- | --- | --- | --- | --- |
|  |  | Species and strain | % similarity | Accession number | e- donor (conc. in mM) | e-acceptor (conc. in mM) |  |  |
| *Actinobacteria* | |  |  |  |  |  |  |  |
|  | *Paraoerskovia* | *Paraoerskovia marina* DSM21750 T | 100 | JNIY01000001 | succinate (2.08mM)/ethanol (4.17mM)/glycerol (2.78mM) | nitrate (3mM)/nitrite (2mM) | 2 | R-66634, R-66635 |
| *Alphaproteobacteria* | |  |  |  |  |  |  |  |
|  | *Labrenzia* | *Labrenzia alba* CECT 5094 T | 99.07 | AJ878875 | succinate (2.08mM)/ethanol (4.17mM)/glycerol (2.78mM) | nitrate (3mM)/nitrite (2mM) | 6 | R-66636, R-66637,  R-66638, R-66639,  R-66640, R-66641 |
|  |  | *Labrenzia aggregata* IAM 12614 T | 99.86 | AAUW01000037 | pyruvate (4.17mM)/acetate (6.25mM) | nitrate (5mM) | 3 | R-52697, R-52691,  R-52692 |
|  | *Martelella* | *Martelella endophytica* YC6887 T | 97.82-98.80 | HM800924 | succinate (10.42mM)/ethanol (20.84mM)/glycerol (13.89mM) | nitrate (3mM)/nitrite (2mM) | 5 | R-66642, R-66643,  R-66645, R-66646,  R-66647 |
|  |  |  |  |  | pyruvate (4.17mM)/acetate (6.25mM) | nitrate (5mM) | 4 | R-52687, R-52690,  R-66644, R-66648 |
|  | *Celeribacter* | *Celeribacter baekdonensis* L-6 T | 98.7-100 | HM997022 | glucose (4.17mM) | nitrate (5mM) | 4 | R-52661, R-52662,  R-52663, R-52664 |
|  |  |  |  |  | glucose (20.83mM) | nitrate (3mM)/nitrite (2mM) | 4 | R-52665, R-52666,  R-52667, R-52668 |
|  |  |  |  |  | 1/10 marine broth | nitrate (5mM) | 1 | R-52651 |
|  | *Pelagicola* | *Pelagicola litorisedimines* D1-W8 T | 99.4 | KC708867 | 1/10 marine broth | nitrate (5mM) | 3 | R-52656, R-52658,  R-52660 |
|  | *Phaeobacter* | *Phaeobacter gallaeciensis* BS107 T | 99.0-99.4 | ABIF01000020 | pyruvate (4.17mM)/acetate (6.25mM) | nitrate (5mM) | 3 | R-52693, R-52695,  R-52698 |
|  | *Loktanella* | *Loktanella rosea* Fg36 T | 99.34 | AY682199 | 1/10 marine broth | nitrate (5mM) | 1 | R-52657 |
|  | *Pseudoruegeria* | *Pseudoruegeria lutimaris* HD-43 T | 98.34 | FJ374173 | 1/10 marine broth | nitrate (5mM) | 1 | R-52653 |
|  | *Roseovarius* | *Roseovarius gaetbuli* YM-20 T | 100 | KF208688 | 1/10 marine broth | nitrate (5mM) | 2 | R-52654, R-52655 |
|  | *Thalassospira* | *Thalassospira lucentensis* DSM 14000T | 99.58 | AM294944 | pyruvate (4.17mM)/acetate (6.25mM) | nitrate (3mM)/nitrite (2mM) | 1 | R-52699 |
|  |  |  |  |  | pyruvate (4.17mM)/acetate (6.25mM) | nitrate (5mM) | 1 | R-52913 |
| *Gammaproteobacteria* | |  |  |  |  |  |  |  |
|  | *Oceanisphaera* | *Oceanisphaera donghaensis* BL1 T | 99.77 | DQ190441 | succinate (10.42mM)/ethanol (20.84mM)/glycerol (13.89mM) | nitrate (5mM) | 2 | R-52674, R-52676 |
|  | *Marinobacter* | *Marinobacter vinifirmus* FB1 T | 99.73 | DQ235263 | succinate (2.08mM)/ethanol (4.17mM)/glycerol (2.78mM) | nitrate (5mM) | 1 | R-52700 |
|  | *Shewanella* | *Shewanella colwelliana* ATCC 39565 T | 100 | AY653177 | 1/10 marine broth | nitrate (5mM) | 2 | R-52649,R-66649 |
|  |  | *Shewanella marisflavi* SW 117 T | 100 | AY485224 | succinate (10.42mM)/ethanol (20.84mM)/glycerol (13.89mM) | nitrate (5mM) | 2 | R-52673, R-52675 |
|  | *Citrobacter* | *Citrobacter gillenii* CDC 4693-86 T | 99.93 | AF025367 | succinate (10.42mM)/ethanol (20.84mM)/glycerol (13.89mM) | nitrate (3mM)/nitrite (2mM) | 1 | R-52910 |
|  | *Shigella* | *Shigella flexneri* ATCC 29903 | 97.7 | X96963 | pyruvate (4.17mM)/acetate (6.25mM) | nitrate (3mM)/nitrite (2mM) | 2 | R-52920, R-52921 |
|  | *Halomonas* | *Halomonas denitrificans* M29 T | 98.96 | AM229317 | pyruvate (4.17mM)/acetate (6.25mM) | nitrate (3mM)/nitrite (2mM) | 1 | R-52914 |
|  | *Vibrio* | *Vibrio alginolyticus* NBRC 15630 T | 99.38-99.73 | CP006718 | succinate (2.08mM)/ethanol (4.17mM)/glycerol (2.78mM) | nitrate (5mM) | 6 | R-52677, R-52678,  R-52679, R-52680,  R-52681, R-52682 |
|  |  |  |  |  | pyruvate (4.17mM)/acetate (6.25mM) | nitrate (5mM) | 2 | R-52696, R-52911 |
|  |  |  |  |  | pyruvate (20.83mM)/acetate (31.25mM) | nitrate (5mM) | 4 | R-52915, R-52916,  R-52917, R-52918 |
|  |  | *Vibrio diabolicus* HE800 T | 99.15-99.36 | X99762 | pyruvate (4.17mM)/acetate (6.25mM) | nitrate (5mM) | 4 | R-52683, R-52684,  R-52685, R-52686 |
|  |  |  |  |  | 1/10 marine broth | nitrate (5mM) | 1 | R-52650 |
|  |  | *Vibrio neocaledonicus* NC470 T | 99.43-99.79 | JQ934828 | succinate (10.42mM)/ethanol (20.84mM)/glycerol (13.89mM) | nitrate (5mM) | 4 | R-52669, R-52670,  R-52671, R-52672 |
|  |  |  |  |  | pyruvate (20.83mM)/acetate (31.25mM) | nitrate (5mM) | 2 | R-52688, R-52689 |
|  |  | *Vibrio rumoiensis* S-1T | 100 | AB013297 | pyruvate (20.83mM)/acetate (31.25mM) | nitrate (3mM)/nitrite (2mM) | 1 | R-66650 |
| *Bacteroidetes* | |  |  |  |  |  |  |  |
|  | *Marinifilum* | *Marinifilum flexuosum* CECT 7448 T | 95.49 | HE613737 | 1/10 marine broth | nitrate (5mM) | 1 | R-52652 |
|  | *Formosa* | *Formosa algae* KMMM 3553 | 99.86 | AY228461 | pyruvate (4.17mM)/acetate (6.25mM) | nitrate (5mM) | 1 | R-52912 |
|  | *Lutibacter* | *Lutibacter agarilyticus* KYW566 T | 97.43 | JN864028 | 1/10 marine broth | nitrate (5mM) | 1 | R-52659 |
|  | *Yeousuana* | *Yeousuana aromativorans* GW 1-1 T | 96.84 | AY682382 | pyruvate (20.83mM)/acetate (31.25mM) | nitrate (5mM) | 1 | R-66651 |
| *Firmicutes* | |  |  |  |  |  |  |  |
|  | *Bacillus* | *Bacillus drentensis* LMG 21831 T | 99.58 | AJ542506 | glucose (4.17mM) | nitrate (5mM) | 3 | R-66632, R-66633,  R-66652 |
